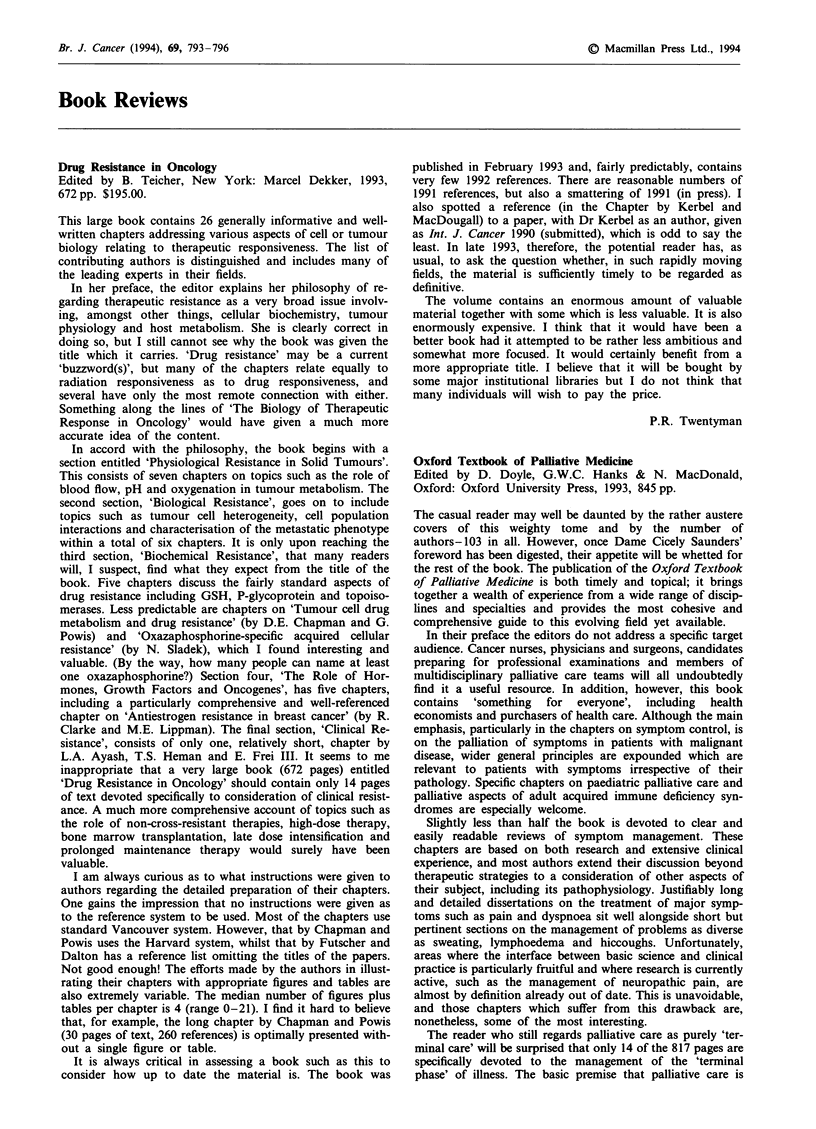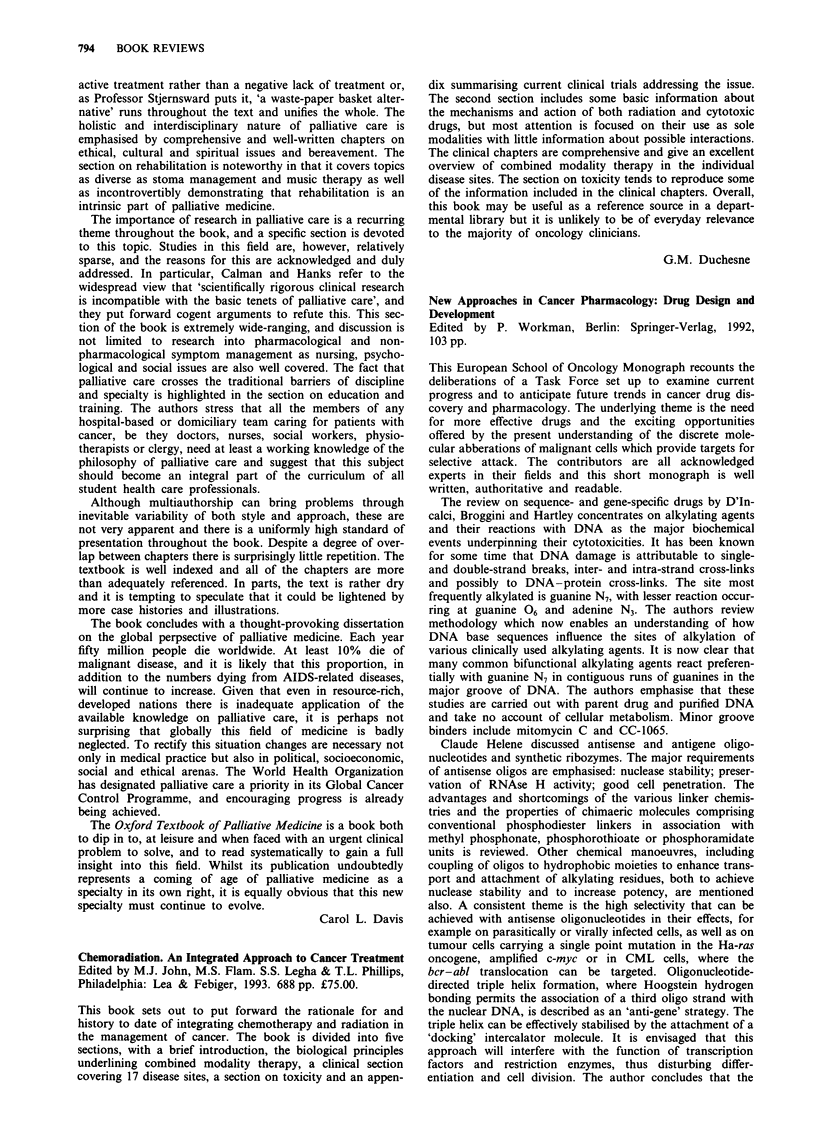# Oxford Textbook of Palliative Medicine

**Published:** 1994-04

**Authors:** Carol L. Davis


**Oxford Textbook of Palliative Medicine**
Edited by D. Doyle, G.W.C. Hanks & N. MacDonald, Oxford: Oxford University Press, 1993, 845 pp.

The casual reader may well be daunted by the rather austere covers of this weighty tome and by the number of authors–103 in all. However, once Dame Cicely Saunders' foreword has been digested, their appetite will be whetted for the rest of the book. The publication of the *Oxford Textbook of Palliative Medicine* is both timely and topical; it brings together a wealth of experience from a wide range of disciplines and specialties and provides the most cohesive and comprehensive guide to this evolving field yet available.

In their preface the editors do not address a specific target audience. Cancer nurses, physicians and surgeons, candidates preparing for professional examinations and members of multidisciplinary palliative care teams will all undoubtedly find it a useful resource. In addition, however, this book contains 'something for everyone', including health economists and purchasers of health care. Although the main emphasis, particularly in the chapters on symptom control, is on the palliation of symptoms in patients with malignant disease, wider general principles are expounded which are relevant to patients with symptoms irrespective of their pathology. Specific chapters on paediatric palliative care and palliative aspects of adult acquired immune deficiency syndromes are especially welcome.

Slightly less than half the book is devoted to clear and easily readable reviews of symptom management. These chapters are based on both research and extensive clinical experience, and most authors extend their discussion beyond therapeutic strategies to a consideration of other aspects of their subject, including its pathophysiology. Justifiably long and detailed dissertations on the treatment of major symptoms such as pain and dyspnoea sit well alongside short but pertinent sections on the management of problems as diverse as sweating, lymphoedema and hiccoughs. Unfortunately, areas where the interface between basic science and clinical practice is particularly fruitful and where research is currently active, such as the management of neuropathic pain, are almost by definition already out of date. This is unavoidable, and those chapters which suffer from this drawback are, nonetheless, some of the most interesting.

The reader who still regards palliative care as purely 'terminal care' will be surprised that only 14 of the 817 pages are specifically devoted to the management of the 'terminal phase' of illness. The basic premise that palliative care is active treatment rather than a negative lack of treatment or, as Professor Stjernsward puts it, 'a waste-paper basket alternative' runs throughout the text and unifies the whole. The holistic and interdisciplinary nature of palliative care is emphasised by comprehensive and well-written chapters on ethical, cultural and spiritual issues and bereavement. The section on rehabilitation is noteworthy in that it covers topics as diverse as stoma management and music therapy as well as incontrovertibly demonstrating that rehabilitation is an intrinsic part of palliative medicine.

The importance of research in palliative care is a recurring theme throughout the book, and a specific section is devoted to this topic. Studies in this field are, however, relatively sparse, and the reasons for this are acknowledged and duly addressed. In particular, Calman and Hanks refer to the widespread view that 'scientifically rigorous clinical research is incompatible with the basic tenets of palliative care', and they put forward cogent arguments to refute this. This section of the book is extremely wide-ranging, and discussion is not limited to research into pharmacological and non-pharmacological symptom management as nursing, psychological and social issues are also well covered. The fact that palliative care crosses the traditional barriers of discipline and specialty is highlighted in the section on education and training. The authors stress that all the members of any hospital-based or domiciliary team caring for patients with cancer, be they doctors, nurses, social workers, physiotherapists or clergy, need at least a working knowledge of the philosophy of palliative care and suggest that this subject should become an integral part of the curriculum of all student health care professionals.

Although multiauthorship can bring problems through inevitable variability of both style and approach, these are not very apparent and there is a uniformly high standard of presentation throughout the book. Despite a degree of overlap between chapters there is surprisingly little repetition. The textbook is well indexed and all of the chapters are more than adequately referenced. In parts, the text is rather dry and it is tempting to speculate that it could be lightened by more case histories and illustrations.

The book concludes with a thought-provoking dissertation on the global perpsective of palliative medicine. Each year fifty million people die worldwide. At least 10% die of malignant disease, and it is likely that this proportion, in addition to the numbers dying from AIDS-related diseases, will continue to increase. Given that even in resource-rich, developed nations there is inadequate application of the available knowledge on palliative care, it is perhaps not surprising that globally this field of medicine is badly neglected. To rectify this situation changes are necessary not only in medical practice but also in political, socioeconomic, social and ethical arenas. The World Health Organization has designated palliative care a priority in its Global Cancer Control Programme, and encouraging progress is already being achieved.

The *Oxford Textbook of Palliative Medicine* is a book both to dip in to, at leisure and when faced with an urgent clinical problem to solve, and to read systematically to gain a full insight into this field. Whilst its publication undoubtedly represents a coming of age of palliative medicine as a specialty in its own right, it is equally obvious that this new specialty must continue to evolve.

Carol L. Davis